# Basal Body Positioning Is Controlled by Flagellum Formation in *Trypanosoma brucei*

**DOI:** 10.1371/journal.pone.0000437

**Published:** 2007-05-09

**Authors:** Sabrina Absalon, Linda Kohl, Carole Branche, Thierry Blisnick, Géraldine Toutirais, Filippo Rusconi, Jacky Cosson, Mélanie Bonhivers, Derrick Robinson, Philippe Bastin

**Affiliations:** 1 Dynamique et Régulation des Génomes, Muséum National d'Histoire Naturelle, INSERM and CNRS, Paris, France; 2 Trypanosome Cell Biology Unit, Pasteur Institute and CNRS, Paris, France; 3 Biologie Fonctionnelle des Protozoaires, Muséum National d'Histoire Naturelle, Paris, France; 4 Biologie du Développement, CNRS, Station zoologique, Villefranche sur Mer, France; 5 Génomique fonctionnelle des Trypanosomatides, Université Bordeaux 2 and CNRS, Bordeaux, France; Dartmouth College, United States of America

## Abstract

To perform their multiple functions, cilia and flagella are precisely positioned at the cell surface by mechanisms that remain poorly understood. The protist *Trypanosoma brucei* possesses a single flagellum that adheres to the cell body where a specific cytoskeletal structure is localised, the flagellum attachment zone (FAZ). Trypanosomes build a new flagellum whose distal tip is connected to the side of the old flagellum by a discrete structure, the flagella connector. During this process, the basal body of the new flagellum migrates towards the posterior end of the cell. We show that separate inhibition of flagellum assembly, base-to-tip motility or flagella connection leads to reduced basal body migration, demonstrating that the flagellum contributes to its own positioning. We propose a model where pressure applied by movements of the growing new flagellum on the flagella connector leads to a reacting force that in turn contributes to migration of the basal body at the proximal end of the flagellum.

## Introduction

Cilia and flagella are prominent organelles whose structure is remarkably conserved from protists to mammals [1]. They are well known for their motility functions and recently sensory roles have been recognised [2]. A flagellum is an extension of a pre-existing organelle, the basal body, a short cylindrical structure related to centrioles and made of 9 triplets of microtubules [3]. It is constructed by polarised addition of new subunits at the distal end of the growing structure, in a process governed by intraflagellar transport (IFT) [4], [5]. Axoneme precursors are loaded on IFT ‘rafts’ composed of ∼15 proteins, transported towards the tip of the flagellum by kinesin II and unloaded at the distal end for incorporation [6]. The modified cargo is transported back towards the basal body by the action of a specific dynein complex. IFT is critical for flagellum formation but also involved in signal transmission from the cilium to the cell body [7].

One important issue in cilia and flagella function is their correct location at the cell surface [8]. This relies on positioning of the basal body as shown in ciliated epithelial cells where hundreds basal bodies are produced *de novo* at the cell centre and migrate at the apical face of the membrane. The actin network is essential for correct migration and positioning of the basal bodies, but the actual motors are unknown [9].

Trypanosomes are uniflagellated protozoa that represent excellent models to study the flagellum, as they can easily be cultivated and are amenable to reverse genetics [1]. *Trypanosoma brucei* is the parasite responsible for sleeping sickness and hence is the most investigated. During its life cycle, it alternates between mammalian hosts (bloodstream stages) and the tsetse fly insect vector (such as the procyclic stage), with significant morphogenetic changes including flagellum positioning [10]. The flagellum exits from the flagellar pocket and is attached to the cell body for most of its length, with the exception of the distal tip. At the site of attachment, a specialised zone of the cortex is recognised, called the flagellum attachment zone or FAZ [11]. It consists of a single filament and of four specialised microtubules associated to the smooth endoplasmic reticulum, both structures initiating close to the flagellar pocket and terminating at the anterior end of the cell body [12]. The basal body of the flagellum is flanked by an immature pro-basal body and both are linked to the kinetoplast, a network of condensed DNA present in the single mitochondrion. Extensive migration of the basal body takes place during flagellum assembly [13]. This is a microtubule-dependent process, although the actual mechanisms are unknown [14]. A small structure called flagella connector (FC) maintains the distal tip of the new flagellum in contact with the side of the mature flagellum [15]. The FC progressively moves towards the distal tip of the old flagellum as the new flagellum elongates [16]. It has been proposed that FC would impose positioning of the new flagellum on the pattern of the existing flagellum, thereby defining cellular organisation [15].

These cellular aspects make trypanosomes a potent model to study basal body positioning. Our recent data suggest that the new flagellum could participate to basal body migration and hence define its own positioning. Inhibition of new flagellum assembly by inducible RNAi silencing of components of the IFT machinery resulted in poor migration of the newly duplicated basal body whereas cortex elongation and nuclear mitosis were unaffected [17]. In this paper, we demonstrate the involvement of flagellum formation, connection and movement in basal body positioning during the trypanosome cell cycle. RNAi silencing of specific flagellum and basal body components was used to inhibit different flagellum-related processes and basal body positioning and cytoskeletal organisation were investigated at both the qualitative and quantitative level. The results demonstrate that basal body and hence flagellum positioning is tightly controlled by a combination of flagellar and cytoskeletal elements.

## Results

### Basal body migration is not linked to cortex elongation

**Figure 1:**
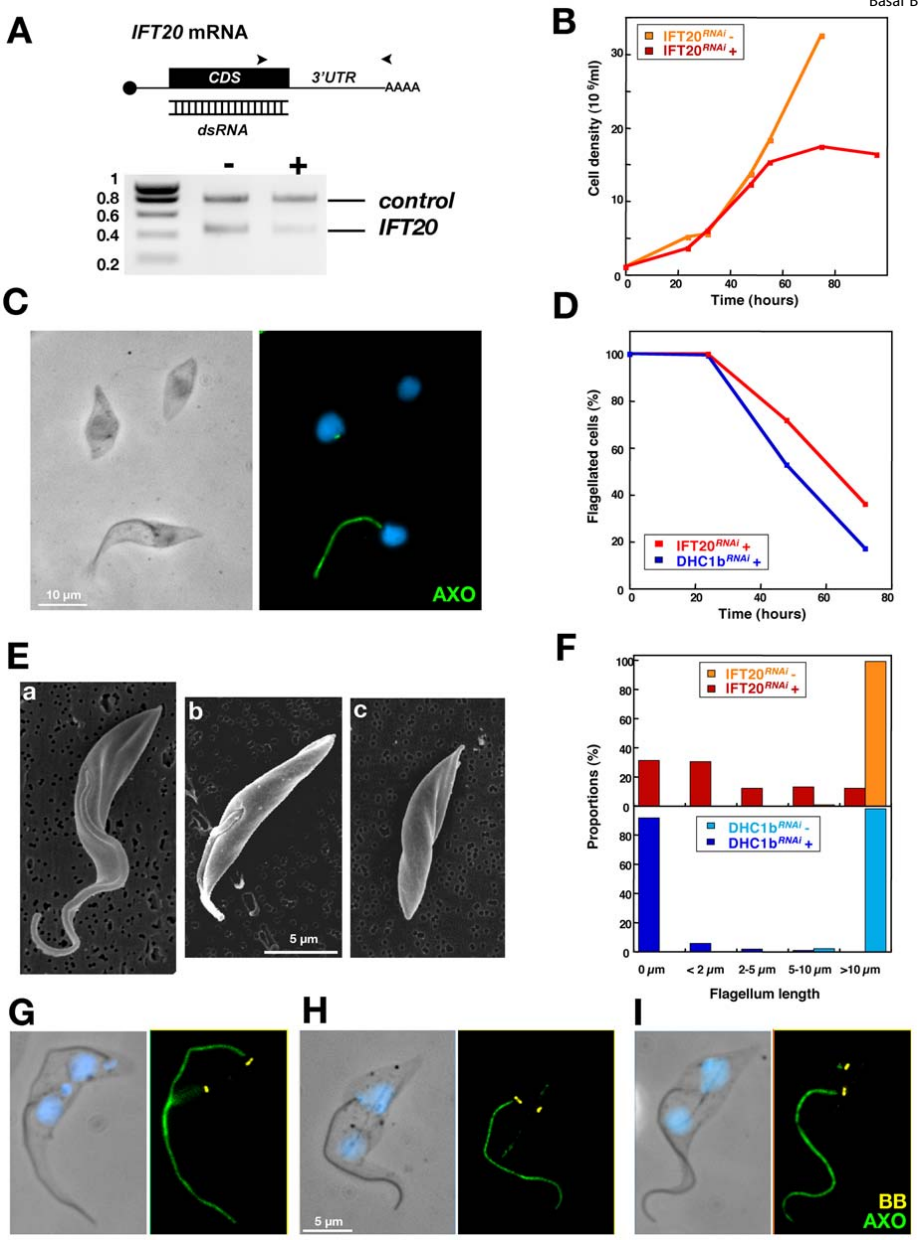
*IFT20* is required for flagellum formation and for correct basal body migration. A. RT-PCR using aldolase and *IFT20* primers (positions indicated by arrowheads) on total RNA from IFT20*^RNAi^* cells grown with (+) or without (−) tetracycline for 72 hours, showing *IFT20* RNA silencing. B. Growth curve of induced (+) and non-induced (−) IFT20*^RNAi^* cells. C. Field of IFT20*^RNAi^* cells induced for 72 h and stained with MAb25 (axoneme [AXO] marker, green) and DAPI (blue) showing cells without flagella or with a flagellum of shorter length. D. Proportion of cells with a flagellum during induction of RNAi silencing in IFT20*^RNAi^* or in DHC1b*^RNAi^* (extent of *DHC1b* RNAi knock-down has been previously demonstrated [17]) E. Scanning electron micrographs of IFT20*^RNAi^* cells induced for 72 h illustrating three kinds of cells: with a normal flagellum (a), with a short flagellum (b) and without flagellum at all (c). F. Length of the new flagellum (when present) in bi-nucleated cells of the indicated cell lines. Cells were grouped in categories according to the indicated flagellar length. G–I. Bi-nucleated cells from non-induced (G) and 72h-induced IFT20*^RNAi^* cells (H–I) stained with MAb22 (basal body marker [BB], yellow), MAb25 (axoneme [AXO] marker, green) and DAPI (blue).

During flagellum and FAZ formation, the new basal body migrates by ∼5 µm, a conspicuous feature of the trypanosome cell cycle (Fig. S1). This extensive movement could be coupled to elongation of the cell cortex, as the distance between the new basal body and the posterior end of the cell is fixed during the cell cycle [13]. Two cell lines expressing double-stranded RNA of *IFT* genes under the control of tetracycline-inducible promoters were selected for thorough analysis of the role of the flagellum in this process: *DHC1b^RNAi^* [17] and *IFT20^RNAi^* (Fig. 1). IFT20 is a component of the IFT particle and is required for cilium assembly in mammalian cells [18]. Silencing of *IFT20* also blocks flagellum formation in *T. brucei* and reproduces the typical phenotype of inhibition of IFT in trypanosomes (Fig. 1). The new flagellum is not assembled resulting in growth arrest (Fig. 1B) exactly as observed previously for other *IFT* genes [17], [19]. The *IFT20^RNAi^* cell line was selected because it exhibits slower kinetics (Fig. 1D,F), producing mixtures of cells with short flagella or no flagella (Fig. 1C–F). Basal body positioning relative to the posterior end of the cell was examined in non-induced (Fig. 1G) and in induced *DHC1b^RNAi^* and *IFT20^RNAi^* cell lines (Fig. 1H–I). Bi-nucleated cells were selected for analysis as they are at the end of their cell cycle, where basal body separation reaches its maximum prior to cell division (Fig. S1). The nuclear cycle is not affected by inhibition of flagellum formation and can thus serve as a marker of the position in the cell cycle for *IFT^RNAi^* cell lines [17]. To evaluate the primary phenotypes due to inhibition of flagellum assembly, only cells that still possessed the old flagellum were analysed (see Materials & Methods). Flagellum and basal bodies were visualised after double staining with specific monoclonal antibodies. MAb22 recognises an as yet unidentified antigen found in both the mature and the pro-basal body, labelling two spots in interphase cells and four in cells assembling a new flagellum (Fig. S1 & Fig. 1G–I)[20]. The flagellum is clearly visible upon detergent-extraction of the cytoskeleton and its axoneme can be decorated by MAb25 that detects a proline-rich protein present on the axoneme of both old and new flagella, staining one or two lines in mono-and bi-flagellated cells respectively (Fig. S1 & Fig. 1G–I)[20]. To evaluate the role of flagellum presence in basal body positioning, several cellular parameters were investigated and quantified. New and old flagellum length, inter basal body distance and position of the new basal body relative to the posterior end of the cell were measured in control cells and during the course of RNAi silencing (Table 1). Basal body segregated correctly in *IFT20^RNAi^* cells as long as their new flagellum had a normal length (Table 1, 24 and 48 h after induction of RNAi). By contrast, when the length of the new flagellum was reduced, the inter basal body distance dropped from 5 to 2.5 µm and the distance between the basal body and the posterior end of the cell increased from 4 to 6 µm (Table 1, 72 h). This became even more obvious in cells that fail to assemble a flagellum in both *IFT20^RNAi^* and *DHC1b^RNAi^* cells (Fig. 1H & Table 1). These results demonstrate that presence of the new flagellum is required for correct positioning of the posterior basal body and that basal body migration is not directly linked to cortex elongation.

**Table 1:** Flagellum length, basal body migration and positioning in binucleated cells

Cell line	Induction time	Flagellum length		Basal body migration	Basal body posterior
		Old	New		
	*h*	*µm*	*µm*	*µm*	*µm*
IFT20*^RNAi^*	0	17.7±1.7	14.2±1.8	5.1±0.9	4.4±0.9
IFT20*^RNAi^*	24	18.0±1.3	14.0±1.6	5.0±0.9	4.1±0.6
IFT20*^RNAi^*	48	18.2±1.8	13.7±1.8	4.8±0.7	4.0±0.5
IFT20*^RNAi^*	72^a^	15.9±4.3	3.5±4.7	2.5±1.6	6.2±1.8
IFT20*^RNAi^*	72^b^	14.9±3.5	0.0	1.9±1.1	6.6±1.8
DHC1b*^RNAi^*	48	16.9±5.1	0.2±0.8^c^	1.9±1.2	6.6±1.6
TBBC*^RNAi^*	48	21.7±2.6	15.1±4.1	2.0±1.4	6.9±2.1
TBBC*^RNAi^*	72	21.9±2.2	15.5±5.4	1.7±0.8	7.3±2.1
PF16*^RNAi^*	48	17.0±2.6	12.3±2.7	2.0±1.2	6.7±1.4
PF20*^RNAi^*	48	20.1±2.3	15.7±2.9	2.2±1.2	6.4±1.3
WT	-	19.8±2.0	16.1±2.3	4.9±0.9	3.3±0.9
*snl*-2	0	20.4±0.9	18.8±1.5	5.2±0.6	3.4±0.5
*snl*-2	10	20.1±1.2	17.9±1.9	5.2±0.7	3.6±0.6
*snl*-2	48	20.3±1.1	17.5±1.1	4.8±0.8	3.3±0.4

Flagellum length was measured using Mab25 staining for the IFT20*^RNAi^* cell lines and using phase contrast image of the flagellum on detergent extracted cells for all other cell lines. As MAb25 does not stain the transition zone, this implies that these measurements are shorter of about 2 µm. A minimum of 100 cells was measured as described in material & methods.

a cells with and without new flagellum

b cells without a new flagellum only, n = 34

c only 8% of bi-nucleated cells possessed a new flagellum

### FAZ restricts basal body migration in the absence of a new flagellum

**Figure 2:**
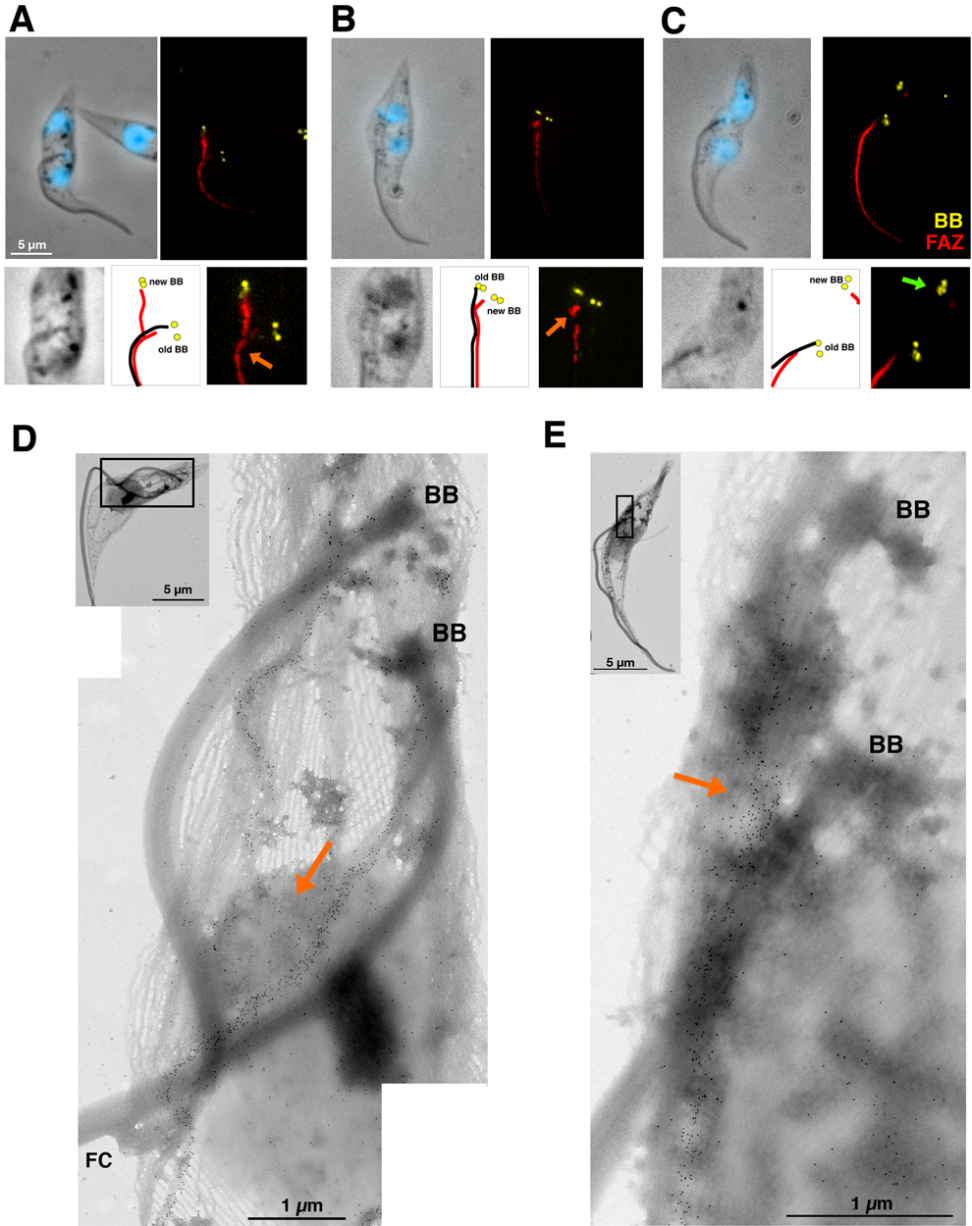
FAZ restricts basal body migration. A–C. Top panels, detergent-extracted cytoskeletons of DHC1b*^RNAi^* cells induced for 41 h stained with MAb22 (basal body marker [BB], yellow), L3B2 (FAZ marker, red) and DAPI (blue). Bottom panels: 2-fold magnification of the basal body area of the above images. Diagrams show the position of the old (with a flagellum) and new (without a flagellum) basal bodies as well as positioning of the flagellum (black lines) and the FAZ filament (red lines). DAPI has been omitted from phase contrast images to facilitate visualisation of the flagellar structures. A–B. Presence of a short new FAZ contacting the old one (orange arrows) appears to refrain new basal body migration. C. Extensive basal body migration when the new FAZ is not in contact with the old one (green arrow). D–E. Detergent-extracted cytoskeletons of non-induced (D) or 48h-induced (E) DHC1b*^RNAi^* cells stained with L3B2 (immunogold) showing interaction between new and old FAZ (orange arrows). Basal bodies (BB) are found at the proximal end of the flagella (when present) and are easily recognised by their thicker wall (due to the presence of triplet microtubules [12]).

**Figure 3:**
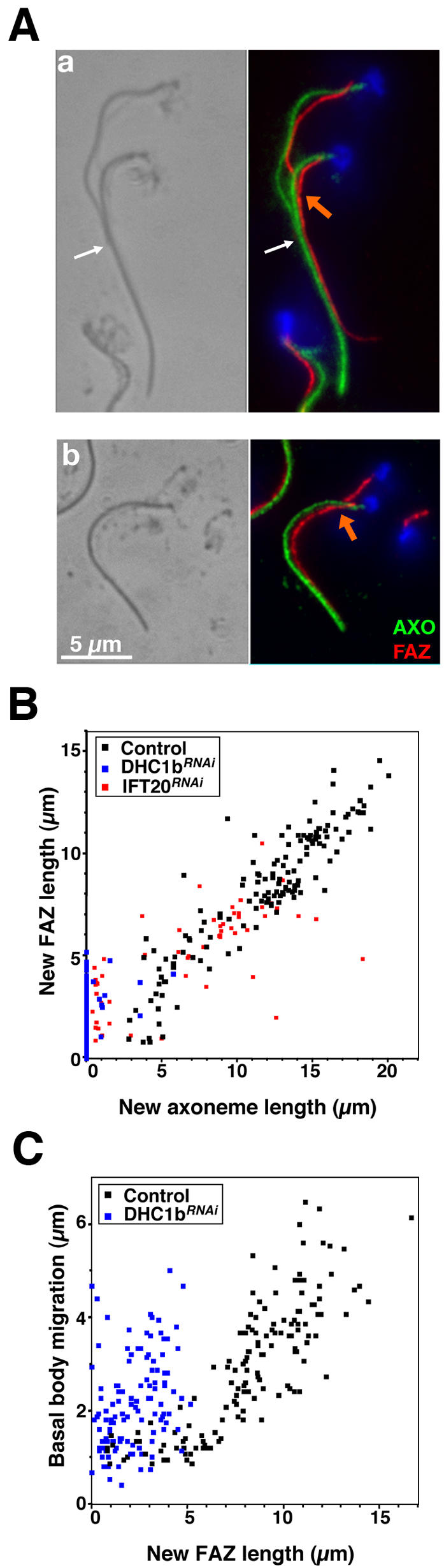
FAZ restricts basal body migration. A. Non-induced (a) or 48h-induced (b) DHC1b*^RNAi^* cells treated with calcium, leading to de-polymerisation of the microtubule corset but not of the FAZ filament. Samples were stained with L6B3 (FAZ marker, red), KMX (anti-tubulin, axoneme [AXO] marker, green) and DAPI (blue). Contacts between old and new FAZ filaments are visible (orange arrow), even in the absence of a new flagellum. The position of the FC on the non-induced cell (top panels) is indicated by a white arrow. B. Length of the new FAZ relative to that of the new flagellum in the indicated cell lines (n>100). FAZ length was measured using the DOT-1 or the L3B2 monoclonal antibody in IFT20*^RNAi^* and DHC1b*^RNAi^* cells respectively. Please note that spots corresponding to IFT20*^RNAi^* cells without a flagellum are hidden by the corresponding spots from DHC1b*^RNAi^* cells. C. Basal body migration relative to FAZ length in the same cells as in H (excepted for IFT20*^RNAi^* cells where basal body migration was measured in a separate experiment).

If the basal body is not linked to the posterior end of the cell, its positioning defect in the absence of new flagellum could be due to retention by associated structures such as the FAZ filament and/or the four associated microtubules that are less efficiently assembled in IFT mutant cells [17]. The FAZ filament was visualised using two monoclonal antibodies: DOT-1 [21] and L3B2 [22] that each recognise distinct antigens of this structure. FAZ length and disposition were investigated in relation with basal body positioning in *DHC1b^RNAi^* and *IFT20^RNAi^* cells (Fig. 2 & Table 2). A severe reduction of the size of the FAZ filament associated to the new basal body was measured in all IFT RNAi mutants examined (Table 2 & Fig. 3B), showing that proper FAZ formation requires flagellum elongation. As described above, bi-nucleated *DHC1b^RNAi^* cells possessing an old but no new flagellum were the most informative as they correspond to early stages of RNAi silencing. In this situation, bi-nucleated cells could be grouped in two categories (Fig. 3C): the most abundant ones (74.5%, n = 122) show an inter-basal body distance inferior to 2.5 µm (Fig. 2A–B) while the remaining 25.5% displayed more extensive separation (Fig. 2C). Immunofluorescence (Fig. 2A–B) and immuno-electron microscopy (Fig. 2E) revealed that when basal body migration was limited, the short new FAZ filament looked attached to the old one. This possible connection could restrict basal body movement and explain the poor basal body migration. Immunogold labelling of the FAZ filament on detergent-extracted cytoskeletons of normal cells (wild-type or non-induced) confirmed these data by revealing that the distal tip of the new FAZ makes contact with the side of the old FAZ (Fig. 2D). That was also observed in *DHC1b^RNAi^* cells with a short new FAZ (Fig. 2E). To evaluate whether this contact point is actually a connection, the microtubule cytoskeleton was de-polymerised by the addition of calcium. This treatment disrupts all microtubules of the corset but does not affect those of the axoneme [22]. In these conditions, the FAZ filament remains intact and can be easily detected by the monoclonal antibody L6B3 that recognises the same antigen as L3B2 [22] whereas axoneme microtubules are visualised with the anti-tubulin antibody KMX [23] (Fig. 3A). In control cells, the images reveal a clear connection between the tip of the new FAZ and the side of the old that is separate from the flagella connection (Fig. 3Aa). However, this could be imposed by the close proximity of the connected flagella. Analysis of *DHC1b^RNAi^* cells lacking the new flagellum but still possessing an old one demonstrated that the new FAZ was formed and linked to the old FAZ (Fig. 3Ab).

**Table 2:** New and old FAZ length in binucleated cells

Cell line	Induction time	FAZ length	
		Old	New
	*h*	*µm*	*µm*
WT	-	15.7±1.7	11.3±2.2
IFT20*^RNAi^*	72	11.3±3.3	4.4±2.2
IFT20*^RNAi^* ^a^	72	11.5±5.9	2.8±1.7
DHC1b*^RNAi^*	48	13.4±3.3	1.9±1.3
TBBC*^RNAi^*	48	16.6±2.7	3.9±4.1^b^
TBBC*^RNAi^*	72	15.9±2.5	2.1±1.7^c^
PF16*^RNAi^*	48	12.0±3.3	4.5±2.5
PF20*^RNAi^*	48	14.7±2.5	5.8±2.7

FAZ length was measured using L3B2 or DOT-1 staining on detergent extracted cells. A minimum of 100 cells was measured as described in material & methods.

a Cells without new flagellum (n = 34)

b Median: 1.8 µm

c Median: 1.6 µm

We next investigated the remaining 25.5% of cells where basal body separation was superior to 2.5 µm. Strikingly, the vast majority of these possessed a short new FAZ filament that clearly lacked contact with the old FAZ structure (Fig. 2C). In some situations, no new FAZ was visible and basal body migration was extensive (data not shown). We conclude that in the absence of a new flagellum, FAZ anchoring restricts basal body migration.

The length of the new flagellum was measured on detergent-extracted cells at all stages of flagellum formation (in both mono-and bi-nucleated cells). FAZ filament length was measured using staining with either L3B2 or DOT-1 (Fig. 3B). In control cells, FAZ length is directly correlated with flagellum elongation as previously shown by comparison with measurement of PFR elongation [22]. However, the PFR is not present on the proximal part of the flagellum [24]. Our analysis shows that for flagella of shorter length, the linear relationship is partially lost and indicates that assembly of the new flagellum is initiated prior to FAZ formation (Fig. 3B). In *DHC1b^RNAi^* cells induced for 48 hours, most cells do not form a new flagellum [17] and their advancement in the cell cycle was judged by MAb22 staining to check whether the basal bodies had duplicated. As the new flagellum is virtually absent from most cells, this means that all spots are limited to the Y axis and reveal that the assembled FAZ filament is very short (below 5 µm). An intermediate situation is observed for *IFT20^RNAi^* cell line induced for 3 days, where a mixture of cells with a shorter flagellum or without flagella were visible (Fig. 1F). In this case, exclusively bi-nucleated cells were analysed. The data reveal that cells that assemble a short new flagellum (<5 µm) also fail to elongate a normal FAZ (please note that spots corresponding to *IFT^RNAi^* cells without new flagellum are obscured by the large number of similar *DHC1b^RNAi^* cells on the graph). By contrast, cells that assemble a flagellum longer than 5 µm construct a FAZ filament of the expected length (Fig. 3B).

Due to the importance of the FAZ filament in the control of basal body migration, we also plotted the inter-basal body distance versus FAZ length in wild-type and in induced *IFT^RNAi^* mutant cells (Fig. 3C). This showed an apparently biphasic process with first a slow migration of basal bodies, followed by a rapid separation for longer FAZ lengths (see below).

### Flagella connection is essential for basal body migration

**Figure 4:**
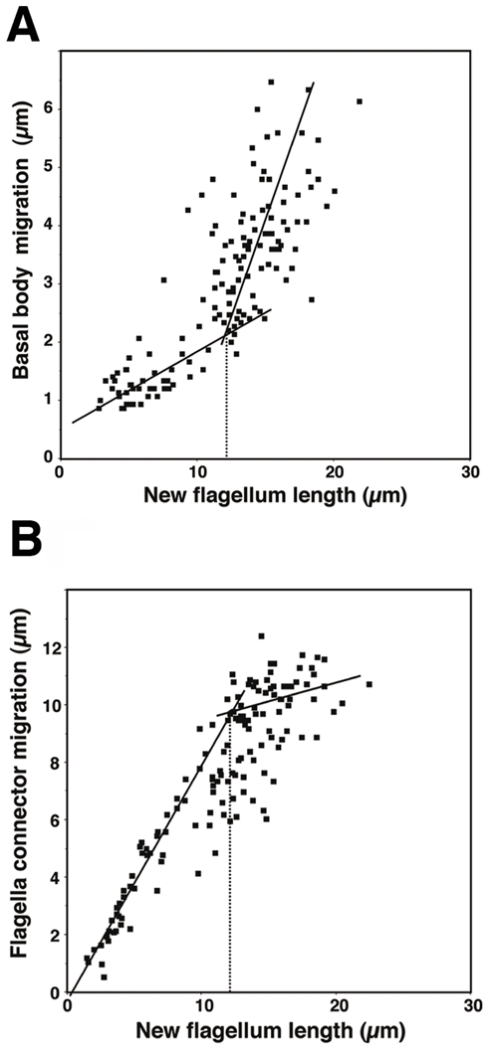
FC migration along the old flagellum is bimodal. A–B. New flagellum length, inter basal body distance and position of the FC on the old flagellum were measured in bi-flagellated wild-type cells at any stage of flagellum assembly.

If FAZ restricts basal body migration, the role of the elongating new flagellum could be to counteract this effect. We previously proposed that flagellum elongation could apply a pressure on the FC that would result in a reaction force due to FC anchoring and lead to basal body movement at the other end of the flagellum [1]. However, the FC is mobile and shifts along the old flagellum [16]. Could a mobile structure provide a good platform ? To address this issue, FC migration and basal body separation were measured during flagellum elongation in wild-type cells (Fig. 4). Basal body migration was biphasic as previously demonstrated [13], with a slow separation until the flagellum reaches ∼two thirds of its length followed by a rapid migration (Fig. 4A). Strikingly, FC migration also turned out to be biphasic: a rapid first phase followed by an almost arrested status (Fig. 4B). Such a relationship was also reported in a recent study by Davidge et al (2006) [19]. Remarkably, the turning point is observed when new flagellum length corresponds to two thirds of the mature flagellum (Fig. 4B). This suggests that when FC migrates rapidly, it cannot provide a firm anchoring point for the flagellum, hence it brings no or minor contribution to basal body separation. This is in agreement with the fact that basal bodies migrate by ∼2 µm in all IFT*^RNAi^* mutants analysed despite the absence of new flagellum (Table 1). By contrast, a fixed FC would offer a good anchoring point to the elongating flagellum, potentially resulting in a reaction force pushing the basal body at the other end.

**Figure 5:**
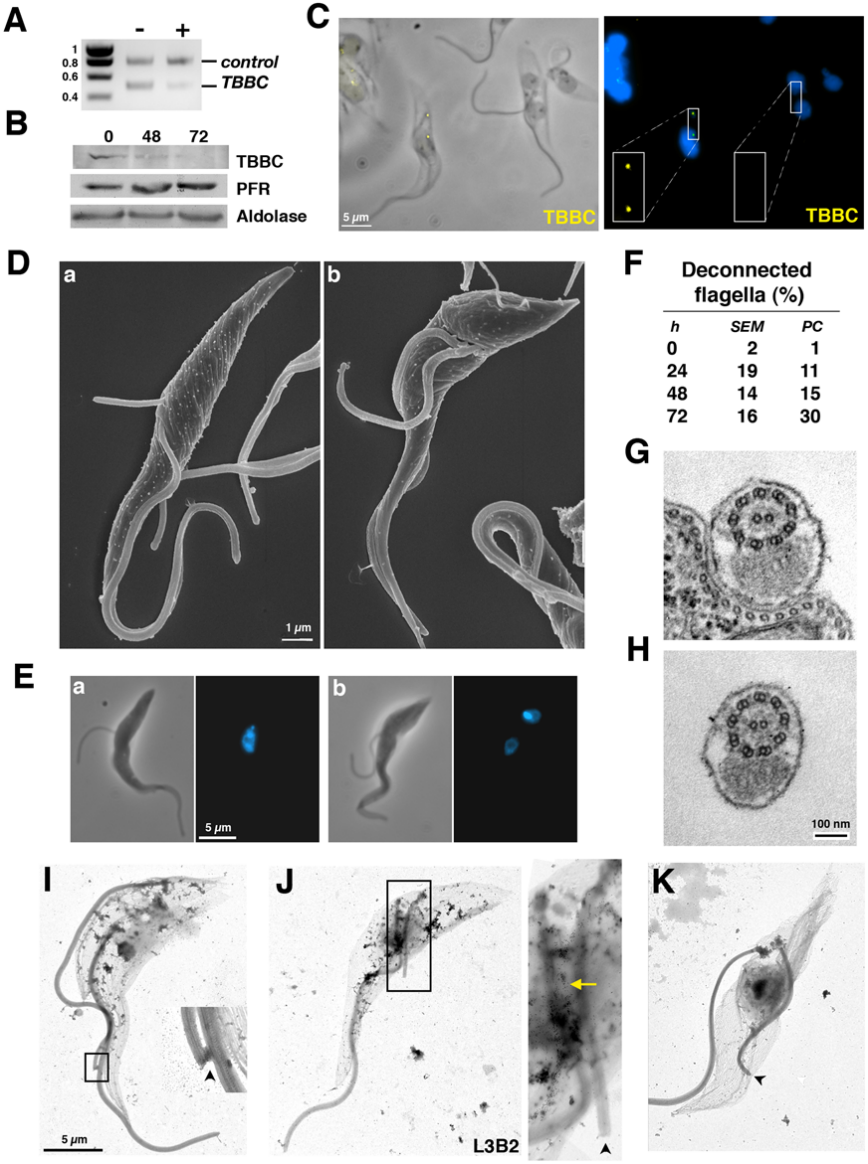
TBBC is necessary for flagella connection. A. RT-PCR using *TBBC* and aldolase primers demonstrates specific *TBBC* RNA silencing as described at Fig. 1A. B. Western blot analysis of TBBC*^RNAi^* cells grown with tetracycline for the indicated periods of time. The same membrane was probed with antibodies against the indicated proteins. C. Immunofluorescence of TBBC*^RNAi^* cells induced for 72 h with the anti-TBBC antibody (yellow) stained with DAPI (blue) showing two bi-flagellated cells, one with normal TBBC staining (left) and one without labelling (right, see insets). Lack of flagella connection is exclusively observed in the latter category. D. Scanning electron micrograph of bi-flagellated cells from TBBC*^RNAi^* cells induced for 48 h. The tip of the new flagellum is free and the flagellum is detached at early (a) and late (b) stages of elongation. E. Glutaraldehyde fixation of similar cells coming from the same population but analysed after DAPI staining. F. The proportion of cells with a deconnected and detached new flagellum (n = 100) during the course of induction (analysed by scanning electron microscopy [SEM] or phase contrast optics [PC]). G–H. Cross-sections of attached (G) or detached (H) flagella from TBBC*^RNAi^* cells induced for 72 h revealing similar ultra-structural organisation. I–K. Detergent-extracted cytoskeletons of 72h-induced TBBC*^RNAi^* cells. When the new flagellum is still connected to the old (I), a clear FC structure is visible (arrowhead). By contrast, no FC is recognised when the new flagellum is not connected, no matter flagellum length (J–K). J. L3B2 staining revealing interactions between old and new FAZ (yellow arrow).

Disruption of the FC could bring a definitive proof of this model. Unfortunately, none of the molecular components of this small structure have been reported to date. However, in a separate project, numerous genes encoding putative flagellum and basal body proteins were silenced (our unpublished data) and one mutant displayed absence of flagellar connection (Fig. 5). The gene silenced encodes TBBC (Trypanosome Basal Body Component), a coiled-coil rich protein mainly localised to the basal body [25]. The TBBC protein is tightly associated to the cytoskeleton, as demonstrated by cell fractionation in detergent (Fig. S2A) where TBBC in a similar way to PFR proteins [26]. Extensive *TBBC* RNAi silencing was shown by RT-PCR (Fig. 5A) and protein ablation by Western blotting (Fig. 5B). A rabbit polyclonal antibody raised against bacterially-expressed TBBC protein [25] was used for immunofluorescence analysis. Immunofluorescence demonstrated that the new flagellum was disconnected from its old counterpart exclusively when TBBC was not detected (Fig. 5C, right). Neighbouring cells that still expressed TBBC were normal with both old and new flagella connected to each other (Fig. 5C, left; Fig. S2B). Both western blot and immunofluorescence results also confirm the specificity of the anti-TBBC antibody. Lack of connection is observed as early as the flagellum exits from the flagellar pocket (Fig. 5Da–Ea). Flagellum elongation then continues normally but the distal tip remains free throughout the rest of the cell cycle (Fig. 5C–4Db–4Eb). The new flagellum is not attached to the cell body, suggesting a role for flagella connection in flagellum adhesion (Fig. 5C–E). Quantification of bi-flagellated cells lacking connection revealed a weaker penetrance of the phenotype (Fig. 5F) compared to other RNAi cell lines. This is not due to RNA silencing which appears as efficient (compare Fig. 5A with Fig. 1A) but could be due to concentration of TBBC at the basal body, meaning that even low amounts of TBBC protein could be detected and possibly sufficient for their function in flagella connection. This actually provides a good control for phenotypic analysis as a mixture of cells with easily recognisable normal and mutant phenotypes is present in the same samples (Fig. 5C). Detached and disconnected flagella beat vigorously (Movie S1, Supplemental Material) and cross sections showed normal axoneme and PFR ultra-structure (Fig. 5G–H) . Measurements of unconnected new flagella in bi-nucleated cells revealed a normal length compared to controls, indicating that elongation is not altered (Table 1). The FC was clearly present and easily recognisable by electron microscopy in control or in induced *TBBC^RNAi^* cells that retain flagella connection and flagellum attachment (14 cells out of 15, Fig. 5I). By contrast, no FC structure was recognisable in cells with an unconnected new flagellum (7 cells out of 7, flagellum of any length; Fig. 5J,K). These differences are not due to variation in sample preparation as cells with connected and unconnected flagella are present on the *same* grid. In conclusion, TBBC is not involved in flagellum assembly or motility but is critical for correct construction (or stabilisation) of the FC. This is the first actual demonstration that FC is indeed required for flagella connection.

**Figure 6:**
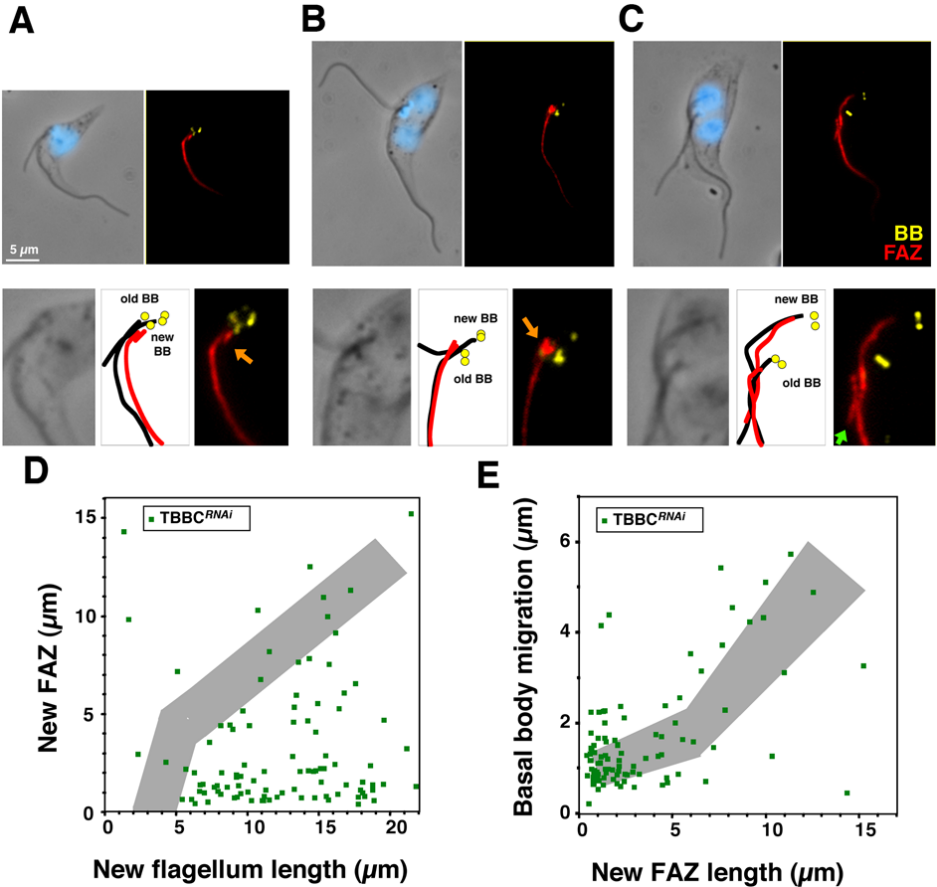
FAZ formation and basal body migration are severely perturbed in the absence of flagella connection. A–C. Top panels: Double labelling of TBBC*^RNAi^* detergent-extracted cells induced for 72 h stained with MAb22 (basal body marker [BB], yellow), L3B2 (FAZ marker, red) and DAPI (blue). Bottom panels: 2-fold magnification of the basal body area of the above images. Diagrams show the position of the old and new basal bodies, as well as positioning of the flagellum (black lines) and the FAZ filament (red lines). A–B. Cells with a short FAZ in close proximity to the old one (orange arrows) have a very limited basal body migration. C. A rare cell with a longer FAZ and normal basal body migration. Note the absence of contact between the tip of the new FAZ and the old FAZ filament (green arrow). D. FAZ length (measured by using the L3B2 signal) is reduced in the absence of flagella connection. Grey shows the range for controls cells (data from Fig. 2). E. Basal body migration compared to FAZ elongation in the same cells.

Examination of live or fixed cells revealed that the unconnected new flagellum was not positioned properly even at late stages of flagellum elongation. A typical example is shown on the right panel of Fig. 5D where the base of the old flagellum and of the new flagellum are in close proximity (Fig. 5D, right panel). This is strongly suggestive of basal body migration defects and was confirmed by immunofluorescence using the MAb22 basal body marker (Fig. 6A–C). The inter basal body distance dropped to less than 2 µm in bi-nucleated cells with unconnected flagella and the distance between the posterior basal body and the tip of the cell also increased to reach more than 7 µm (Table 1). As observed for IFT mutants, cell elongation at the posterior end and nuclear mitosis were unaffected in cells at this stage of induction (Fig. 6). FAZ assembly is dramatically reduced in the absence of FC, with less than 5% of biflagellated cells displaying a filament of normal length (Table 2 & Fig. 6D–E). By contrast, cells that still possessed TBBC signal at the basal body looked normal (flagella connection and attachment, basal body migration and FAZ formation, Fig. 5C & S2B). These data indicate that the FC or flagellum adhesion (or both) are essential for proper FAZ elongation and could serve as guide for its construction. As was observed for the *DHC1b^RNAi^* mutant (Fig. 2A–C), the short new FAZ filament appears to maintain the new basal body in vicinity of the old one (Fig. 5J & 5A–B). In the rare situations where cells with unconnected flagella were seen with normal basal body migration, the new FAZ was not in contact with the old one (Fig. 6C). In conclusion, flagella connection is required for basal body migration in the presence of a shorter new FAZ linked to the old FAZ.

### Base-to-tip flagellum movement is required for basal body migration

**Figure 7:**
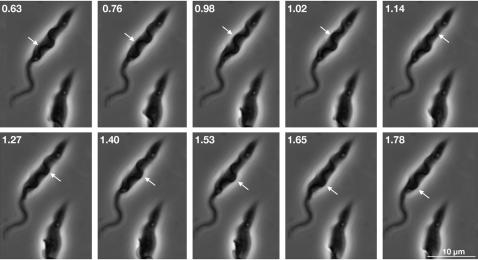
The new flagellum displays both tip-to-base and base-to-tip movements. Still images of Movie S2 (Supp. Mat.). Numbers indicate time in seconds and hundredths of seconds. Arrows indicate propagation of one wave bend (from tip to base on the first 4 images, from base to tip for the other ones).

In control trypanosomes, the new flagellum is motile as it exits from the flagellar pocket. Could this motility contribute to the reaction force model ? Uniflagellated trypanosomes use a tip-to-base wave propagation system, leading to forward motility with the flagellum leading. This is sometimes interrupted by waves propagating from base-to-tip, resulting in cell re-orientation rather than backward motility [27]. Bi-flagellated cells were analysed by video-microscopy, revealing that the new flagellum alternates between episodes of waves propagating from tip to base and waves propagating from base to tip (Movie S2 & Fig. 7). Waves propagating from tip to base tended to be mostly (but not exclusively) of low amplitude and high frequency whereas those propagating from base to tip often displayed high amplitude and low frequency. Intriguingly, the tip of the new flagellum seems to be shifted in different directions along the old flagellum when waves alternate. It moved towards the distal end of the old flagellum during transitions to base-to-tip movement of the new flagellum and towards the proximal end during transitions to tip-to-base movement (Movie S3). This could explain the dispersion seen in FC position of cells with long flagella (Fig. 4B). We reasoned that waves transmitted from the base to the tip of the new flagellum could apply a pressure on the FC and hence actively contribute to basal body migration. This can be investigated due to the existence of mutants with defects in different aspects of beating [27].

Silencing of PFR2, a structural component of the paraflagellar rod, blocks construction of this extra-axonemal structure and results in severe reduction of motility [26]. Such cells show rapid alternation between tip-to-base and base-to-tip waves, without net forward motility [27]. Flagellum length, inter-basal body distance and basal body positioning relative to the posterior end were measured in bi-flagellated cells with the anti-PFR2 monoclonal antibody L8C4 [22] allowing direct monitoring of PFR2 presence. Situations where only the new or where both the new and the old flagella were deprived of PFR2 were analysed and revealed no significant modification of basal body positioning at any stage of the cell cycle (Fig. S3 & Table 1). Flagellum length was not altered by the absence of a PFR and basal body migration occurred normally at all stages of PFR2 silencing (Fig S3 & Table 1). This indicates that when both types of waves are present (even if their repartition is modified), basal body migration proceeds normally. We next investigated bi-flagellated cells from the *DNAI1^RNAi^* mutant where the tip-to-base wave propagation is completely abrogated but that are still able to generate the base-to-tip wave. Basal body migration was normal, exactly as in the *PFR2^RNAi^* mutant or as in control cells (data not shown), demonstrating that the tip-to-base flagellar wave propagation is not required for this process.

**Figure 8:**
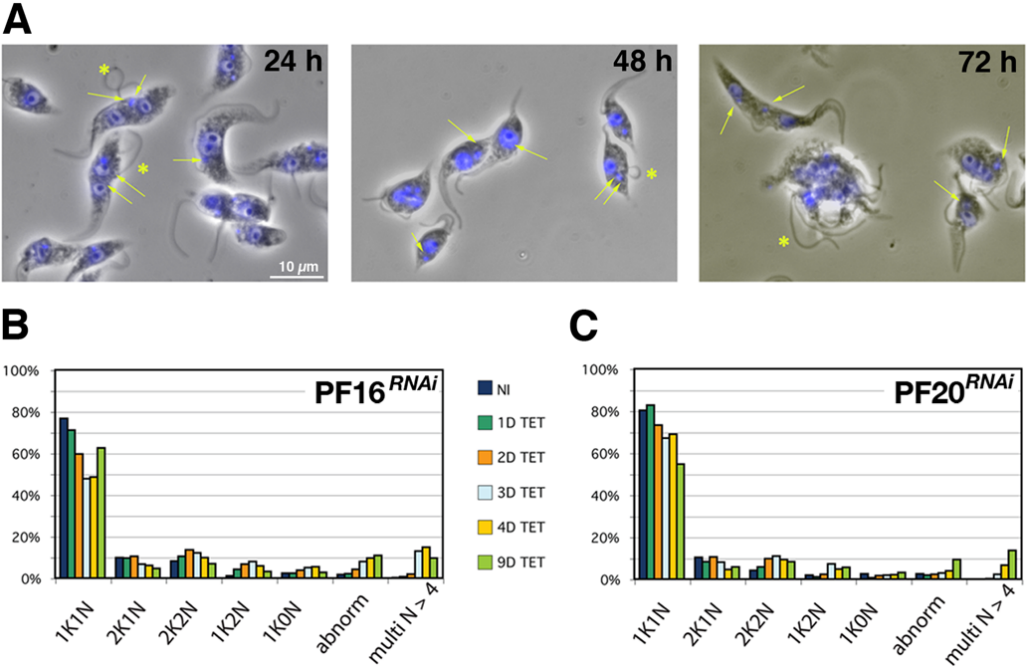
Inhibition of tip-to-base motility leads to multiple cell cycle defects. A. Phase contrast images of methanol-fixed PF16*^RNAi^* cells induced for 24, 48 and 72 h (left to right) stained with DAPI (blue). Arrows point at kinetoplast aberrantly positioned and stars indicate detached flagella. B–C. Distribution of cells according to kinetoplast (K) and nucleus (N) number in PF16*^RNAi^* cells (B) and PF20*^RNAi^* cells (C) grown in the presence of tetracycline for the indicated number of days.

**Figure 9:**
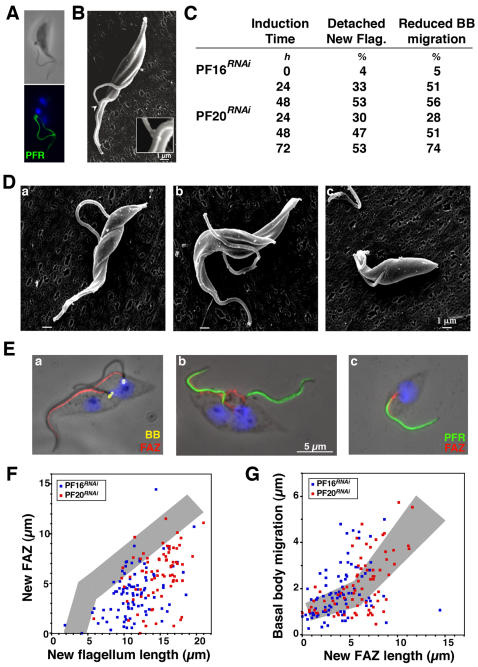
Inhibition of tip-to-base motility results in defects in basal body migration. A. Paraformaldehyde-fixed PF16*^RNAi^* cell induced for 48 h stained with DAPI (blue) and ROD-1 (PFR, green). Only one kinetoplast is visible with two nuclei, two flagella and un-segregated basal bodies. B. Scanning electron micrograph revealing the presence of the FC at the distal end of the detached but not de-connected new flagellum. C. Flagellum attachment and basal body migration in bi-flagellated cells in the indicated cell lines during the course of RNAi silencing (n>50). D–E. PF16*^RNAi^* cells induced for 48 h with a detached new flagellum (a), with a detached and de-connected new flagellum (b) and a uniflagellated cell whose flagellum is not positioned properly (c). D. Scanning electron micrographs. E. Detergent-extracted cytoskeletons stained with MAb22 (basal body [BB] marker, yellow), L3B2 (FAZ marker, red, and DAPI (blue, left panel) or with ROD-1 (PFR marker, that is found in the flagellum as soon as it emerges from the flagellar pocket, green), L3B2 (FAZ marker, red) and DAPI (blue, right panels). F. FAZ length measured using the L3B2 antibody is reduced in PF16*^RNAi^* or PF20*^RNAi^* cells with a detached new flagellum at all stages of flagellum elongation (n>100). Grey shows control cells (see Fig. 2). G. Basal body migration compared to FAZ elongation in the same cells.

A different result was observed upon silencing of PF16 or PF20 that encode proteins of the central pair. This leads to potent block of base-to-tip and tip-to-base waves and to a severe reduction of cell motility [27], [28]. This motility phenotype is accompanied by cell shape defects, flagellum detachment and mis-positioning of the basal body (Fig. 8A)[27]. These parameters were first quantified in uni-flagellated cells, revealing a rapid increase in the frequency of these defects during the course of PF16 and PF20 RNAi silencing (Table 3). The proportion of cells with an abnormal basal body position rose rapidly to reach ∼70% of the population in both cell lines 3 days after RNAi induction. The basal body was often found above the nucleus or even in a position anterior to the nucleus (Fig. 8A, see also Fig. 9E). In such situations, cells were frequently shorter with a shape reminiscent to that of the non-flagellated cells observed during the IFT RNA silencing experiments [17](Fig. 1), suggestive of defects in positioning of the axis of cell cleavage. Moreover, the flagellum was partially or completely detached from the cell body (with the exception of its anchoring point at the basal body) in 60–75% cells, a feature that was tightly correlated with the defects in basal body positioning (Table 3).

**Table 3:** Phenotype of PF16*^RNAi^* and PF20*^RNAi^* in uniflagellated cells

Cell line	Induction time		Flagellum			Wrong cell shape	Aberrant basal body position
			Attached	Detached	Partially		
	*h*	*n*	*%*	*%*	*%*	*%*	*%*
PF16*^RNAi^*	0	500	95	4	1	3	5
PF16*^RNAi^*	24	501	92	8	0	3	19
PF16*^RNAi^*	48	423	19	38	43	75	67
PF16*^RNAi^*	72	322	26	34	40	56	67
PF20*^RNAi^*	24	500	98	2	0	2	8
PF20*^RNAi^*	48	461	83	17	0	14	33
PF20*^RNAi^*	72	397	51	22	27	36	76

Flagella were considered as detached when they were only anchored to the cell *via* the basal body. All intermediate situations were scored as partially detached (only a portion of the flagellum attached to the cell body). When the basal body was found on top of the nucleus or in a position anterior to the nucleus, it was scored as aberrant.

We analysed the cell cycle by DAPI staining to visualise nuclear (N) and kinetoplast (K) DNA content (Fig. 8). Kinetoplast duplication precedes that of the nucleus in trypanosomes, defining three main categories of cells with 1K1N, 2K1N and 2K2N [12](see also Fig. S1 for links with the flagellum cycle). The proportion of each of these cell types, in addition to any other cells with unusual DNA content was quantified during the course of RNAi induction (Fig. 8). The most obvious modification was an increase in multi-nucleated cells due to retardation of cell cleavage, because of the participation of flagellum movement in the final step of cell separation [27], [28]. Another feature was the accumulation of cells with one kinetoplast and two nuclei (∼10% of the population). As the kinetoplast replicate first, these cells are very rare in normal populations (<1%). These 1K2N cells were hence examined more closely. In *PF20^RNAi^*, 72% of them (n = 45) were in fact bi-flagellated cells with a large kinetoplast (Fig. 9A), indicating failure in basal body migration. Observation of 2K2N cells confirmed that basal body separation was reduced and this appeared linked to detachment of the new flagellum (Fig. 9A–C). This migration defect was further documented by measuring basal body migration in bi-nucleated cells that still possessed a normal old flagellum attached to the cell body but whose new flagellum was detached (Table 1)(Fig. 9D–E). This revealed poor migration of the new basal body associated to increased distance between the new basal body and the posterior end of the cell, exactly as observed during silencing of *IFT* genes and of *TBBC* (Table 3). These migration defects led to situations where the posterior basal body was found above the nucleus (Fig. 9Ea) or even between the two nuclei (Fig. 9Eb). These configurations are conserved in daughter cells (Fig. 9Ec), leading to situations where both flagella are detached at the next cycle with complex and aberrant cell shapes (not shown).

When the new flagellum is detached from the main cell body, its distal tip is frequently connected to the old flagellum, and the FC is clearly present (Fig. 9B, Fig. 9Da–8Ea). However, in some bi-flagellated and bi-nucleated cells, the tip of the new flagellum is not connected to the old one, with the new flagellum totally free from the cell body with the exception of its anchoring point (Fig. 9Db–Eb). By contrast to TBBC*^RNAi^* cells, this was exclusively seen at late cell cycle stages when the connection has to be severed for division.

FAZ formation in *PF16^RNAi^* and *PF20^RNAi^* cells was investigated using L3B2 staining, and clearly revealed a reduction in its elongation in bi-flagellated cells where the new flagellum was detached from the cell body (Fig. 9E–F). This inhibition was less severe compared to induced TBBC*^RNAi^* or IFT*^RNAi^* cells (Table 2). The distal tip of the new FAZ appears in contact with the old FAZ in most bi-flagellated cells analysed (>230 cells, Fig. 9Ea–b). FAZ filament length was compared with the inter-basal body distance (Fig. 9G), showing that when the new FAZ is short, basal body migration is limited and if the new FAZ is long, basal body separation appears to proceed reasonably well. In conclusion, base-to-tip motility is required for basal body migration and full FAZ elongation.

## Discussion

### A model for flagellum-dependent basal body migration

Basal body migration is biphasic in procyclic *T. brucei* [13]. This work reveals that the new flagellum contributes to the second, most extensive, phase of basal body positioning by a combination of at least three factors: elongation, connection to the existing flagellum and base-to-tip wave propagation. Individual inhibition of any of these parameters reduces basal body separation to ∼2 µm in all mutants analysed without interfering with the nuclear cycle or cell elongation. This reduction in basal body migration is always linked to defects in construction of the new FAZ filament that is present but too short (only 2–5 µm even at late stages of the cell cycle instead of 11–12 µm for controls). As the proximal end of the new FAZ is associated to the new basal body and since its distal end is linked to the old FAZ, formation of a FAZ that is too short restricts basal body migration. In normal cells too, the tip of the new FAZ is linked to the old FAZ, suggesting that flagellum function in basal body migration would be to counteract this retention. This is coherent with the fact that new basal bodies lacking a FAZ filament are free to migrate even in the absence of a new flagellum and can be found close to the posterior end (Fig. 2C, 5C). These conclusions are supported by qualitative and quantitative (more than 4,000 individual cells were measured in this study) analysis of several mutants: *DHC1b^RNAi^* and *IFT20^RNAi^* (this was also observed in 10 other RNAi mutants where flagellum formation had been inhibited, our unpublished data); *TBBC^RNAi^*, *PF16^RNAi^* and *PF20^RNAi^*. Remarkably, basal body migration takes place normally in *DNAI1^RNAi^* and *PFR2^RNAi^* cells that are still exhibiting base-to-tip movement in their new flagellum or in other RNAi cell lines where various genes not linked to the flagellum are silenced (our unpublished data).

We propose the following model to explain the contribution of the flagellum to basal body migration. Trypanosomes duplicate their basal bodies and construct their new flagellum whose tip is anchored to the old one by the FC and the tip of the new FAZ filament is linked to the old one. The FC migrates slowly as the new flagellum elongates to reach a length of 12 µm and basal bodies remain in close proximity to allow completion of kinetoplast duplication [13]. FC migration on the old flagellum is independent of the new flagellum as it is observed in its absence [17], [19]. Once the new flagellum grows beyond 12 µm, FC migration stops and base to tip waves in the new flagellum exert a longitudinal force on the FC. As the FC is tightly connected to both flagella, this pressure results in a reaction force that is transmitted towards the base of the new flagellum and that leads to displacement of the basal body and possibly contributes to extension of the new FAZ filament. In the absence of new flagellum (silencing of *IFT* genes), without flagella connection (silencing of *TBBC*) or when base-to-tip flagellar waves are inhibited (silencing of *PF16* or *PF20*), this process cannot take place and basal bodies do not migrate extensively whereas FAZ elongation is seriously reduced. As old and new FAZ are linked, failure to elongate the new FAZ hinders basal body movement. In *PF16^RNAi^* or *PF20^RNAi^* cells, both distal (blocked by the FC) and proximal (marked by the basal body) ends of the new flagellum cannot move, explaining that elongation results in detachment. Tip-to-base motility or actual cell swimming are not required for basal body migration, as seen in DNAI1*^RNAi^* and PFR2 *^RNAi^* mutants. RNAi silencing of the dynein-regulatory complex protein trypanin leads to aberrant motility and partial flagellum detachment but basal body migration has not been investigated [29]. Silencing of the “parkin-coregulated” gene affects microtubule doublet formation, cell motility and basal body migration (although this was not quantified) but analysis of remaining flagellar waves have not been reported [30].

Basal body positioning would be the result of a balance between forces that push the new basal body towards the posterior end of the cell and factors that refrain this migration. In this paper, we show that the flagellum contributes to this movement and that the FAZ filament restricts basal body migration. However, other factors could be involved. The minority of cells with new basal bodies devoid of flagellum and of FAZ connection (Fig. 2C) revealed that such basal bodies are not lost anywhere in the cell but are regularly found close to the posterior end. Analysis of a separate RNAi mutant that produces cells with a short unconnected new flagellum also lacking a new FAZ revealed that the basal body was localised at the far posterior end of the cell (M.B. & D.R., unpublished data). This suggests that as yet unidentified structures or mechanisms could contribute to targeting of the basal body at the far posterior end.

### TBBC and FC formation

A participation of the FC in basal body movement was first suggested by its absence from cell types with limited basal body migration such as bloodstream stages of *T. brucei* or the promastigote stage of *Leishmania* [16]. Consistent with our model, gene knock-out of *DHC1b* in the promastigote stage of *L. mexicana* blocks flagellum formation but does not interfere with basal body positioning [31]. How could TBBC interfere with FC formation ? Immunofluorescence studies exclude the possibility that TBBC is a component of the FC [25]. Given its location at the distal end of the mature basal body, TBBC could participate to the filter role proposed for this region in the control of protein import in the flagellum [32]. In its absence, access of one or more components of the FC to the new flagellum would be reduced resulting in a non-functional FC. This leads to a lack of flagella connection actually demonstrating for the first time the role of FC in flagella connection. This supports an essential function of this structure in cell morphogenesis as initially hypothesised [15].

Cilia guiding formation of other cytoskeletal structures are encountered in other species such as in the development of stereocilia of mouse inner ear whose organisation is controlled by a single cilium (kinocilium) linked *via* specific connections. Mutations in linker proteins results in human genetic diseases marked by disorganised stereocilia [33]. Investigating possible interactions between cilia and other cellular components in various organisms and cell types promises to be interesting.

### Flagellum-dependent basal body migration

The trypanosome flagellum contributes to positioning its own basal body, which is in contrast to several eukaryotic species investigated so far where basal body location is defined prior to axoneme formation [8]. This could be linked to the fact that trypanosomes, like most protists, proliferate while maintaining their existing cilia or flagella [12], [34]–[36]. In these species, the nuclear membrane remains present throughout the cell cycle and mitosis takes place without the help of centrioles. Basal bodies duplicate, new flagella are constructed and accurately distributed between the progeny. In contrast, metazoa separate cell and cilium/flagellum cycles: existing flagella are disassembled before mitosis and the bald basal bodies act as centrioles for nuclear mitosis [3], [37]. This implies that mechanisms that involve the control of the cell cycle are likely to differ if flagella persist or not throughout the cell cycle and comparative work should reveal exciting new features. This is particular significant as several recent studies indicate that perturbation of cilia formation or function observed in several genetic diseases results in drastic modification of the cell cycle [38].

## Materials and Methods

### Trypanosome cell lines and cultures

All cell lines were derivatives of strain 427 of *T. brucei* and cultured in SDM79 medium supplemented with hemin and 10% foetal calf serum. Cell line *snl-2* is a derivative of the PTH cell lines [39] transformed with a vector leading to inducible expression of a hairpin double-stranded RNA of *PFR2* (previously called *PFRA*) [40]. Silencing of PFR2 leads to defects in PFR assembly and absence of cell motility due to continuous alternation between the two types of waves but does not alter the structure of the axoneme. Cell lines DHC1*^RNAi^* [17], PF16*^RNAi^* and PF20*^RNAi^* [27] have previously been described and all express double stranded RNA from two tetracycline-inducible T7 promoters facing each other in the pZJM vector [41] transformed in 29-13 cells that express the T7 RNA polymerase and the tetracycline-repressor [42]. As loss of motility inhibits the final step of cell division [27], [28], cultures of induced PF16*^RNAi^* and PF20*^RNAi^* cells were shaken to avoid accumulation of big cellular aggregates [26]. The complete *IFT20* coding sequence (GeneDB reference number: Tb927.6.3290) was amplified by PCR from wild-type *T. brucei* strain 427 genomic DNA as template using GCCGCGCAAGCTTATGGATGATGATAAACTTGTG as forward primer (*Hin*dIII site underlined) and CGTCGTCTCGAGCTACTCACGCGAGGCGTGACTC as reverse primer (*Xho*I site underlined). Amplified products were digested with *Hin*dIII and *Xho*I and ligated in the corresponding sites of the pZJM vector. A fragment from the *TBBC* coding sequence [25] (GeneDB reference number: Tb927.8.6070) was amplified using GGAAGCGGCGACATCTGAGG as forward primer and CTTCAAGCGCCTTATTGGTC as reverse primer. This product contained internal *Hin*dIII and *Xho*I restriction sites that were used for digestion and ligation in pZJM. Both vectors were separately transformed in 29-13 cells, immediately cloned and all phleomycin-resistant cell lines were characterised by immunofluorescence with the anti-PFR2 antibody L8C4. Clones with the clearest distinction between non-induced and induced samples were selected for sub-cloning by limiting dilution, generating the IFT20*^RNAi^* and TBBC*^RNAi^* cell lines. RNAi was induced by addition of 1 µg tetracycline per ml of medium and fresh tetracycline was added at each cell dilution.

### Immunofluorescence and morphogenetic measurements

For immunofluorescence, intact cells or detergent-extracted cytoskeletons were fixed in methanol and processed as described [12], [43]. As PFR markers, we used the monoclonal antibodies L8C4 (IgG1), that specifically recognises PFR2 [22] and ROD-1, an IgM that recognises a minor component of the distal zone of the PFR [21]. The monoclonal antibody MAb25 (IgG2a) recognises an axonemal protein of unknown function and was employed as axoneme marker [20]. The anti-ß-tubulin antibody KMX [23] was also a good marker for the axoneme and used for analysis of calcium-treated cytoskeletons. It also stains the sub-pellicular microtubules and hence cannot be used on whole cells or detergent-extracted cytoskeletons. L3B2 (IgG1) and L6B3 (IgM) recognise the same antigen in the FAZ filament [22] whereas DOT-1 picks up a different component of this structure [21], but all three antibodies turned out to be good markers of the FAZ filament. Monoclonal MAb22 is an IgM that detects an as yet unidentified antigen found at the proximal zone of both the mature and the pro-basal body (M.B. and D.R., unpublished data)[20]. TBBC presence and localisation was analysed with a rabbit polyclonal antiserum [25]. Sub-class specific secondary antibodies coupled to FITC (Sigma), Alexa 488 (Invitrogen), Cy3 (Jackson) or Cy5 (Jackson) were used for double or triple labelling. Slides were stained with DAPI for visualisation of kinetoplast and nuclear DNA content. Samples were observed with a DMR Leica microscope and images were captured with a Cool Snap HQ camera (Roper Scientific). Images were analysed and cell parameters were measured using the IPLab Spectrum 3.9 software (Scanalytics & BD Biosciences) or Image J . Live cells were observed in their culture medium and images for movies were acquired as described [27].

Inter-basal body distance was measured after immunofluorescence staining with MAb22. As the antibody recognises both mature and pro-basal body, measurements were taken from the spots found to be in continuity with the flagellum (when present). In the case of cells lacking the new flagellum (IFT RNAi cell lines), this criterion cannot be applied and the measurement was made from the point situated exactly at the middle of the two basal bodies. Flagellum length was measured either using the MAb25 axoneme marker or from phase contrast images of detergent-extracted cytoskeletons. This procedure makes the flagellum conspicuous by phase contrast microscopy and previous data revealed that this extraction does not modify significantly the length or positioning of several cytoskeletal components [12], [13], [22]. MAb25 does not stain the transition zone, hence flagellum length measured by this antibody is ∼2 µm shorter (18 µm instead of 20 µm for a normal mature flagellum). Flagellum elongation takes about 5 h to be complete in wild-type trypanosomes [12] and its rate appears constant throughout the cell cycle [39]. FC migration was measured by two methods: staining with an anti-FC rabbit antibody [17] superimposed to the phase contrast image to visualise flagella or by direct determination of the contact point between old and new flagella as seen by phase contrast or by MAb25. Unfortunately, the anti-FC [17] was too weak to be used in double immunofluorescence experiments. Data were analysed using best-fit approaches (Kaleidagraph, Synergy Software). FAZ length was measured using L3B2 in combination with MAb22 in order to obtain data for both FAZ extension and basal body migration in the same cells. Measurements of FAZ length using DOT-1 gave very similar values. Data were presented as a function of flagellum length allowing to cover the entire duration of flagellum growth. Once cells completed mitosis, cell division takes about one hour. This means that bi-nucleated cells cover a short phase of the cell cycle where flagellum elongation should be within a window of 3–4 µm [39]. Bi-nucleated cells were therefore selected for the quantification of flagellum and FAZ length, as well as for inter basal body separation, measurements summarised at Table 1 and 2. As it is difficult to reproducibly synchronise trypanosomes and that RNAi silencing effects are unlikely to have the same consequences according to the stage of the cell cycle the protein is knocked-down, identification of the initial events of silencing is critical. This can directly be analysed in the case where antibodies against the targeted protein are available (TBBC, PFR2) but more difficult to assess for the other situations. However, clear morphological criteria could be applied for IFT cell lines (presence of a normal old flagellum) and for central pair axoneme mutants (presence of an attached old flagellum). Except where indicated, a minimum of 100 images were acquired and employed for quantitative analyses.

### Electron microscopy

Detergent-extracted cytoskeletons were prepared on grids and negatively stained as described [12]. Bound antibodies were visualised by addition of secondary anti-mouse antibody coupled to gold particles (average size 10 nm, BB International). Grids were analysed on a Jeol JEM 1010 electron microscope. For scanning electron microscopy, cells were washed in PBS, fixed for at least 1 h with 2.5% glutaraldehyde in PBS or in cacodylate buffer, washed and post-fixed in OsO4 1%. After dehydration, samples were critical-point dried (Emitech K850 or Balzers Union CPD30) and coated with gold (Jeol JFC-1200 or Gatan Ion Beam Coater 681). Samples were visualised with a Jeol 840A or with a Jeol JM6700 F scanning microscope. For transmission electron microscopy, cell fixation, embedding and sectioning were carried out as described previously [27].

### RT-PCR

Total RNA was extracted from cells grown with or without tetracycline for the indicated periods of time and purified using Trizol. DNA was eliminated by DNase treatment and RNA purity was confirmed by conventional PCR. After primer calibration and determination of optimal conditions, semi-quantitative RT-PCR was performed as described [44]. At least one primer was selected to be outside the region selected for dsRNA expression to avoid amplification of RNA deriving from the dsRNA trigger. Primers AGGAAGCTGAACATCGGCTA (1598-1617 of the coding sequence) and AGAGGGTTTTCTCACGCTCA (2076-2095) were used for *TBBC* and CGGAACTCGACCGTTATA (293-310) or CCGCGCAAAGATGAACTC in combination with CCGGATCCTTTTTTTTTTTTTTTTTTTT for *IFT20* and aldolase respectively. Extent of RNAi silencing for *DHC1b^RNAi^* [17], *PF16^RNAi^* and *PF20^RNAi^* [27] has been demonstrated previously.

### Western blot

Cells were washed in PBS, homogenised and boiled in gel sample buffer before SDS-PAGE separation. For fractionation, cells were washed in PBS, treated with 1% Nonidet P-40 in PEM buffer (Pipes 100 mM, pH 6.9, EGTA 1 mM, MgSO_4_ 1 mM) and detergent-resistant and soluble fractions were separated by centrifugation for 5 minutes at 15,000 *g*. Proteins were transferred to PVDF membranes and incubated with anti-TBBC antibody (dilution 1:1,000) as described [25]. Membranes were probed with the anti-PFR antibody L13D6 or with a rabbit anti-aldolase antibody as loading control and revealed with ECL+(Amersham).

## Supporting Information

Figure S1 The cell cycle of T. brucei at the procyclic stage in culture. Detergent-extracted cytoskeletons of wild-type cells were triple-stained with MAb25 (axoneme marker, [AXO] green), MAb22 (basal body marker [BB], yellow), L3B2 (FAZ filament marker, red) and DAPI (blue). Centre panels: the new basal body is always found at the posterior side of the existing one and the new flagellum elongates with its distal end orientated towards the anterior end of the cell. Right panels: extensive basal body migration is always observed at the late stages of the cell cycle such as after nuclear mitosis.
(0.34 MB JPG)

Figure S2 A. TBBC is exclusively associated to the cytoskeleton in control trypanosomes. Cells were fractionated in detergent and soluble and pellet (corresponding to the cytoskeleton) fractions and analysed by western blotting. The same membrane was probed with the indicated antibodies. B. TBBCRNAi cells non-induced (top) or induced for 72 h (bottom) with an anti-TBBC antibody (yellow) stained with DAPI (blue). The basal body is stained in all cells of non-induced samples whereas cells lacking signal in the induced samples (arrows) show deconnected or detached flagella. Cells that retain TBBC signal at the basal body were not affected (left cells).
(0.28 MB JPG)

Figure S3 Basal body migration is normal after PFR2 silencing. Detergent-extracted cytoskeletons of non-induced snl-2 cells (left panels) or induced to express PFR2 dsRNA for 10 h (centre panels) or 4 days (right panels) double-stained with L8C4 (anti-PFR2, PFR marker, green), MAb22 (basal body marker [BB], yellow) and DAPI (blue). Basal body migration takes place normally at all stages of silencing (with PFR2 missing from the new or from both flagella).
(0.24 MB JPG)

Movie S1 A TBBCRNAi cell induced for 48 hours. The new flagellum is detached and not connected to the old flagellum that still appears normal. Both flagella are motile and show vigorous beating.
(1.08 MB MOV)

Movie S2 Control cell with a new and an old flagellum. The new flagellum alternates between tip-to-base and base-to-tip waves. Still images of this movie are presented at figure 6.
(0.30 MB MOV)

Movie S3 Control cell with a new and an old flagellum. When the new flagellum oscillates between the two types of waves, its distal end appears shifted either towards the distal end of the old flagellum (beginning of this sequence) or towards the proximal end (second part of this sequence).
(0.39 MB MOV)
