# Supplementary figures and images for: Basal Body Positioning Is Controlled by Flagellum Formation in Trypanosoma brucei
Source: PLoS One. 2007 May 9;2(5):e437. doi: 10.1371/journal.pone.0000437 (PMC1857822; doi:10.1371/journal.pone.0000437)

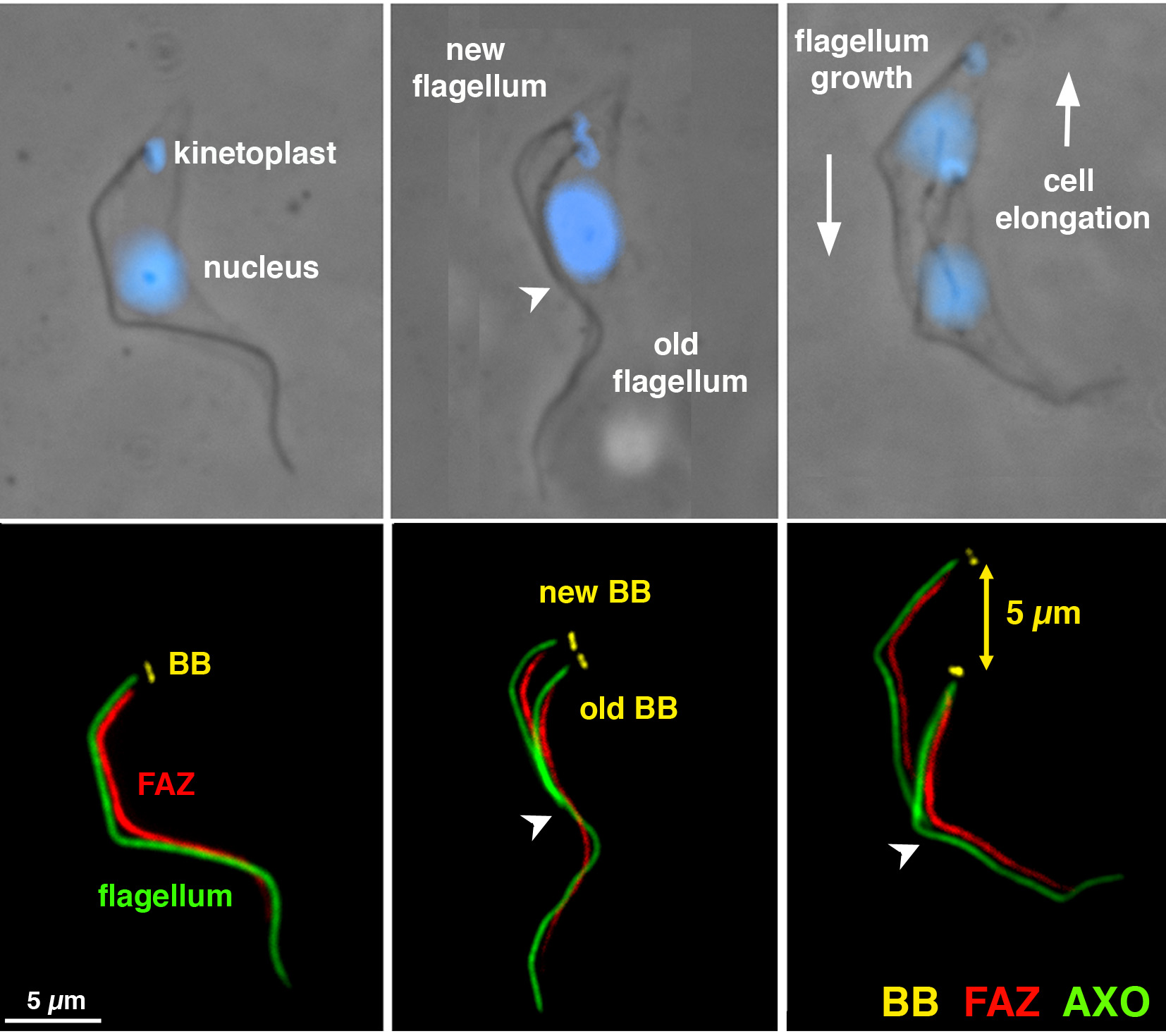

Supplement: Figure S1 — The cell cycle of T. brucei at the procyclic stage in culture. Detergent-extracted cytoskeletons of wild-type cells were triple-stained with MAb25 (axoneme marker, [AXO] green), MAb22 (basal body marker [BB], yellow), L3B2 (FAZ filament marker, red) and DAPI (blue). Centre panels: the new basal body is always found at the posterior side of the existing one and the new flagellum elongates with its distal end orientated towards the anterior end of the cell. Right panels: extensive basal body migration is always observed at the late stages of the cell cycle such as after nuclear mitosis. (0.34 MB JPG) [file pone.0000437.s001.jpg]

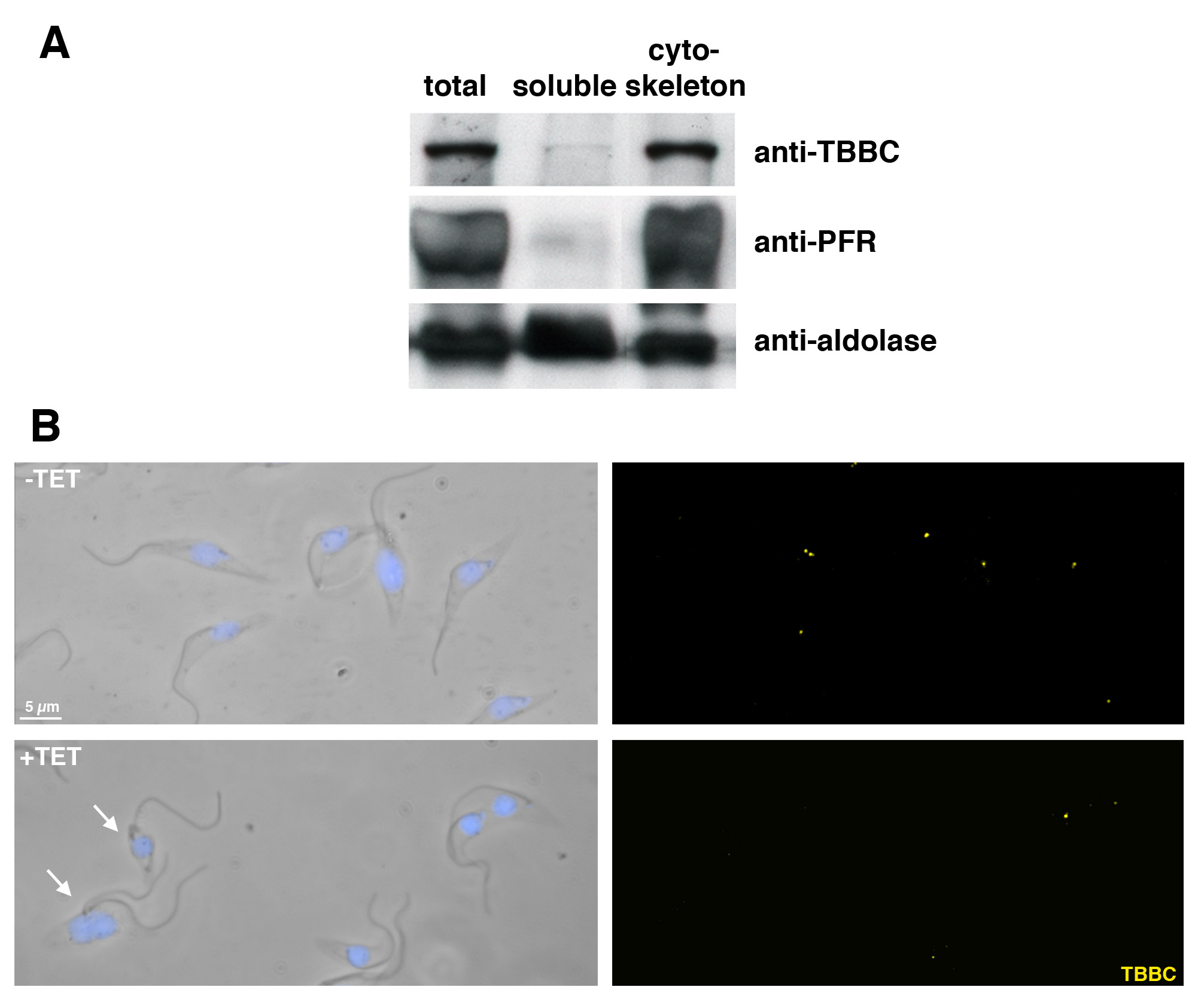

Supplement: Figure S2 — A. TBBC is exclusively associated to the cytoskeleton in control trypanosomes. Cells were fractionated in detergent and soluble and pellet (corresponding to the cytoskeleton) fractions and analysed by western blotting. The same membrane was probed with the indicated antibodies. B. TBBCRNAi cells non-induced (top) or induced for 72 h (bottom) with an anti-TBBC antibody (yellow) stained with DAPI (blue). The basal body is stained in all cells of non-induced samples whereas cells lacking signal in the induced samples (arrows) show deconnected or detached flagella. Cells that retain TBBC signal at the basal body were not affected (left cells). (0.28 MB JPG) [file pone.0000437.s002.jpg]

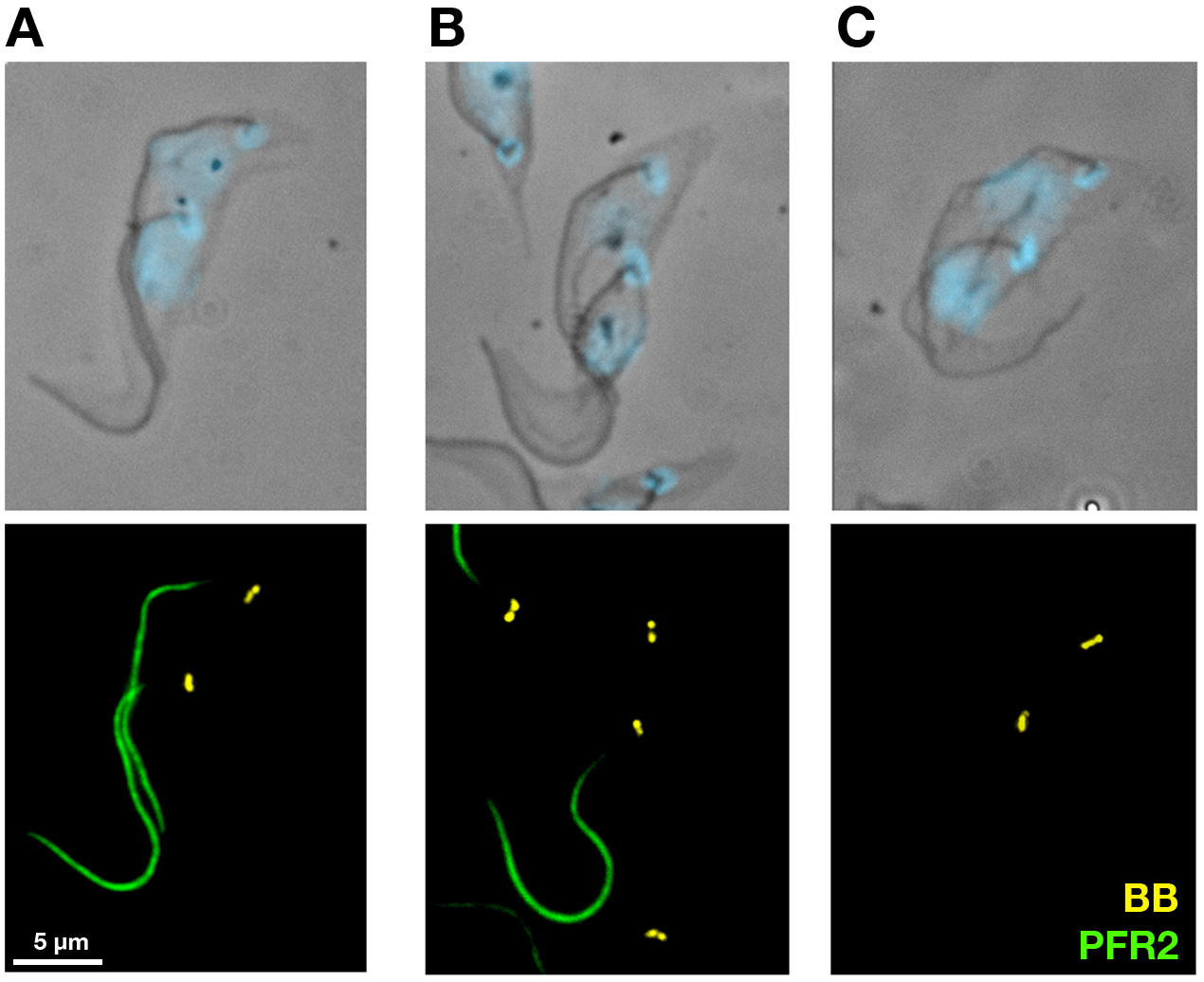

Supplement: Figure S3 — Basal body migration is normal after PFR2 silencing. Detergent-extracted cytoskeletons of non-induced snl-2 cells (left panels) or induced to express PFR2 dsRNA for 10 h (centre panels) or 4 days (right panels) double-stained with L8C4 (anti-PFR2, PFR marker, green), MAb22 (basal body marker [BB], yellow) and DAPI (blue). Basal body migration takes place normally at all stages of silencing (with PFR2 missing from the new or from both flagella). (0.24 MB JPG) [file pone.0000437.s003.jpg]
